# Ecology and distribution of *Leptospira* spp., reservoir hosts and environmental interaction in Sri Lanka, with identification of a new strain

**DOI:** 10.1371/journal.pntd.0010757

**Published:** 2022-09-16

**Authors:** Vincent Sluydts, Siriwardana Rampalage Sarathchandra, Anna Pia Piscitelli, Natalie Van Houtte, Sophie Gryseels, Anne Mayer-Scholl, Nadja Seyhan Bier, Nyo Me Htwe, Jens Jacob

**Affiliations:** 1 Julius Kühn-Institute, Federal Research Institute for Cultivated Plants, Institute for Plant Protection in Horticulture and Forests, Vertebrate Research, Münster, Germany; 2 University of Antwerp, Department of Biology, Evolutionary Ecology Group, University of Antwerp, Wilrijk, Belgium; 3 Rice Research and Development Institute, Batalagoda, Ibbagamuwa, Kurunegala, Sri Lanka; 4 Federal Institute for Risk Assessment, Department of Biological Safety, Berlin, Germany; DotLab, UNITED STATES

## Abstract

Leptospirosis is a neglected zoonotic disease and one of the leading causes of zoonotic morbidity and mortality, particularly in resource-poor settings. Sri Lanka has one of the highest disease burdens worldwide, with occasional endemic leptospirosis outbreaks (2008, 2011). Rodents are considered the main wildlife reservoir, but due to a scarcity of studies it is unclear which particular species contributes to bacterial transmission and reservoir maintenance in this multi-host multi-parasite system. Several rodent species act as agricultural pests both in rice fields and in food storage facilities. To unravel the interactions among the small mammal communities, pathogenic *Leptospira* spp. and human transmission pathways, we collected animals from smallholder food storage facilities, where contact between humans and small mammals is most likely, and screened kidney tissue samples for *Leptospira* spp. using PCR. Samples were collected in three climatic zones along a rainfall gradient. Pathogenic *Leptospira* spp. were detected in small mammal communities in 37 (74%) out of 50 sampled farms and 61 (12%) out of 500 collected individuals were infected. The small mammal community was comprised of *Rattus rattus* (87.6%), *Suncus* shrews (8.8%), *Bandicota* spp. (2.8%) and *Mus booduga* (0.8%). Three pathogenic *Leptospira* spp. were identified, *L*. *borgpetersenii* (n = 34), *L*. *interrogans* (n = 15), and *L*. *kirschneri* (n = 1). *Suncus* shrews were commonly infected (32%), followed by *B*. *indica* (23%) and *R*. *rattus* (10%). *L*. *borgpetersenii* strains similar to strains previously extracted from human clinal samples in Sri Lanka were detected in *R*. *rattus* and *Suncus* shrews. *L*. *interrogans* was observed in *R*. *rattus* only. A single *L*. *kirschneri* infection was found in *M*. *booduga*. The presence of human pathogenic *Leptospira* species in an agricultural pest rodent (*R*. *rattus*) and in commensal shrews (*Suncus*) calls for management of these species in commensal settings. Further investigation of the interplay between pathogen and reservoir population dynamics, overlap in geographic range and the extent of spill-over to humans in and around rural settlements is required to identify optimal management approaches.

## Author summary

Leptospirosis is a bacterial disease which affects livestock and humans, causing symptoms ranging from asymptomatic to severe morbidity and even mortality in Sri Lanka. The bacterium is known to survive well outside the hosts under favourable conditions such as in a rice-ecosystem. In low-income situations the wellbeing of families can suffer significantly if the principal breadwinner is affected or their livestock turns out sick. In this study rodent host species were sampled in and around smallholder rice storage structures in Sri Lanka with the aim of building knowledge on the wildlife reservoir and potential spill-over events. Leptospira were commonly detected in the black rat and in commensal shrew species. Genetic analysis demonstrated that *Leptospira* isolates found in these small mammal species were similar to those already isolated from human clinical cases from Sri Lanka. In a biodiversity hotspot such as Sri Lanka, this information can be used to design and implement management strategies which focus on particular host species.

## Introduction

Rodent-borne diseases can significantly affect humans and livestock with consequences ranging from asymptomatic or mild symptoms, to severe morbidity and even mortality [1]. Public health cost is immense [2] and particularly in low-income situations, the wellbeing of families suffers if the principal breadwinner is sick for an extended period of time. Leptospirosis is a re-emerging zoonotic infectious disease of worldwide distribution with a considerable public health impact, particularly in impoverished populations [3–5]. The causative agent is a spirochaetal bacterium of the genus *Leptospira*, which cycles between multiple host species and the environment [6]. The environmental pool of spirochetes is maintained by persistent excretion through chronically infected hosts, commonly rodents, small mammals, livestock and dogs. Under favourable conditions, the spirochetes can survive for several weeks to months outside the host organism [7]. Transmission occurs when humans are exposed to urine of infected hosts, or through exposure to contaminated water, soil or food. As accidental hosts, humans are not regarded as reservoir for transmission [8]. In tropical regions, an increase in human incidence is often associated with heavy rainfall or flooding [5,9]. Recent morbidity and mortality estimates predict a high disease burden in South and South-East Asian regions [10,11], with rice-ecosystems typically associated to an increased risk for leptospiral infection [6].

In Sri Lanka, human leptospirosis was well documented in the ‘60 and ‘70, but got neglected for nearly four decades until a major outbreak with over 7,000 reported cases in 2008 [12]. This fueled a body of literature on human cases and etiological agents [> 50 research papers since 2008], but only three publications in the last 20 years focused on livestock and rodent species as maintenance reservoir or accidental hosts [13–15]. One publication noted domestic elephants to shed *Leptospira* spp. to the environment, suggestive for a risk of transmission during certain cultural and religious events [16]. In Sri Lanka, rodents such as black rats and bandicoot rats as well as livestock are reported to be infected with *Leptospira* spp. [14] and it was suggested that livestock is a more important source for spill-over to humans [9,14]. Four *Leptospira* species (*L*. *borgpetersenii*, *L*. *kirschneri*, *L*. *weilii* and *L*. *interrogans*) have been isolated from humans in Sri Lanka [17], with *L*. *interrogans* being identified as the principal species associated with leptospirosis [18].

Sri Lanka is well recognized for its high biodiversity. In the order *Rodentia*, family Muridae, sixteen species occur on Sri Lanka of which five are endemic. Given the scarcity of studies on wildlife host of pathogenic *Leptospira* spp. in Sri Lanka, it is unclear how different species of small mammals contribute to parasite transmission and the maintenance reservoir. However, those species occupy different ecological niches, ranging from commensal rodents primarily occurring inside houses and in storage facilities to rodent species occurring mainly in rice-fields or natural habitats. In addition, seasonality may affect the occurrence of different species, some of which will migrate between agricultural fields and human settlements in search of food and shelter.

In this study, we aimed to extend the knowledge about the reservoirs of pathogenic *Leptospira* spp. in Sri Lanka, focusing on the rodent community in and around smallholder food storage facilities. These structures are usually attached to farmer houses or are nearby and could represent an important interface for pathogen transmission between rodents and humans. Therefore, these structures are relevant to both food security and human health because of significant damage and contamination of stored food stuffs [19]. Building knowledge about the wildlife reservoir will help to determine how management strategies originally developed to reduce losses in crop production can affect prevalence and spill-over of important rodent-borne diseases to humans.

## Material & methods

### Ethics statement

The Ethics Review Committee of the Institute of Biology Sri Lanka provided ethical clearance for the project under registration number ERC IOBSL 201 09 2019.

### Site description

**Fig 1:**
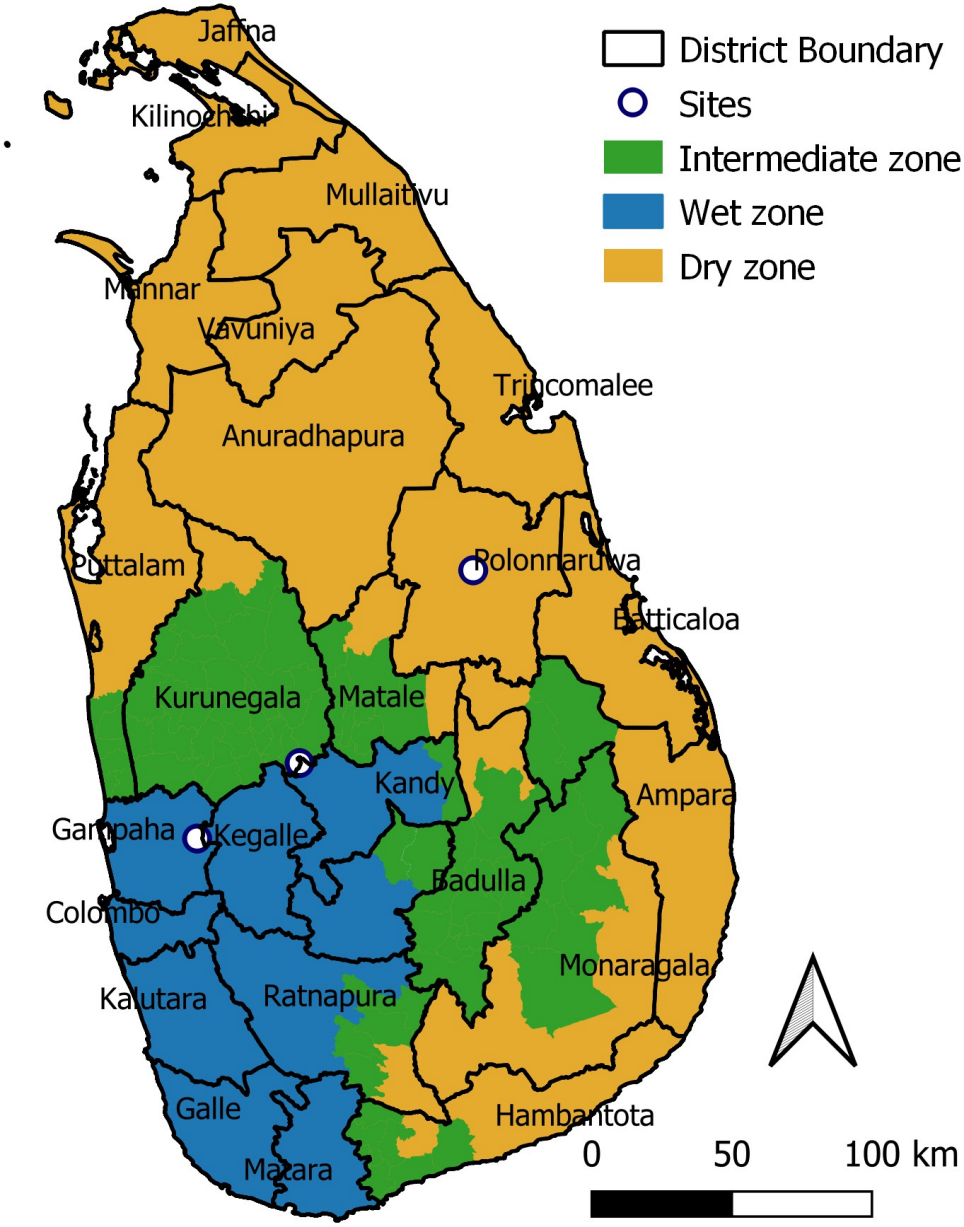
Climate zones according to rainfall, districts and location of sampling sites (Polonnaruwa, Kurunegala and Gampaha) in Sri Lanka where field work was conducted in smallholder food storage facilities. Source base layer & credit base layer: https://data.humdata.org/ published under creative commons attribution for intergovernmental organisations: https://data.humdata.org/m/dataset/cod-ab-lka.

**Fig 2:**
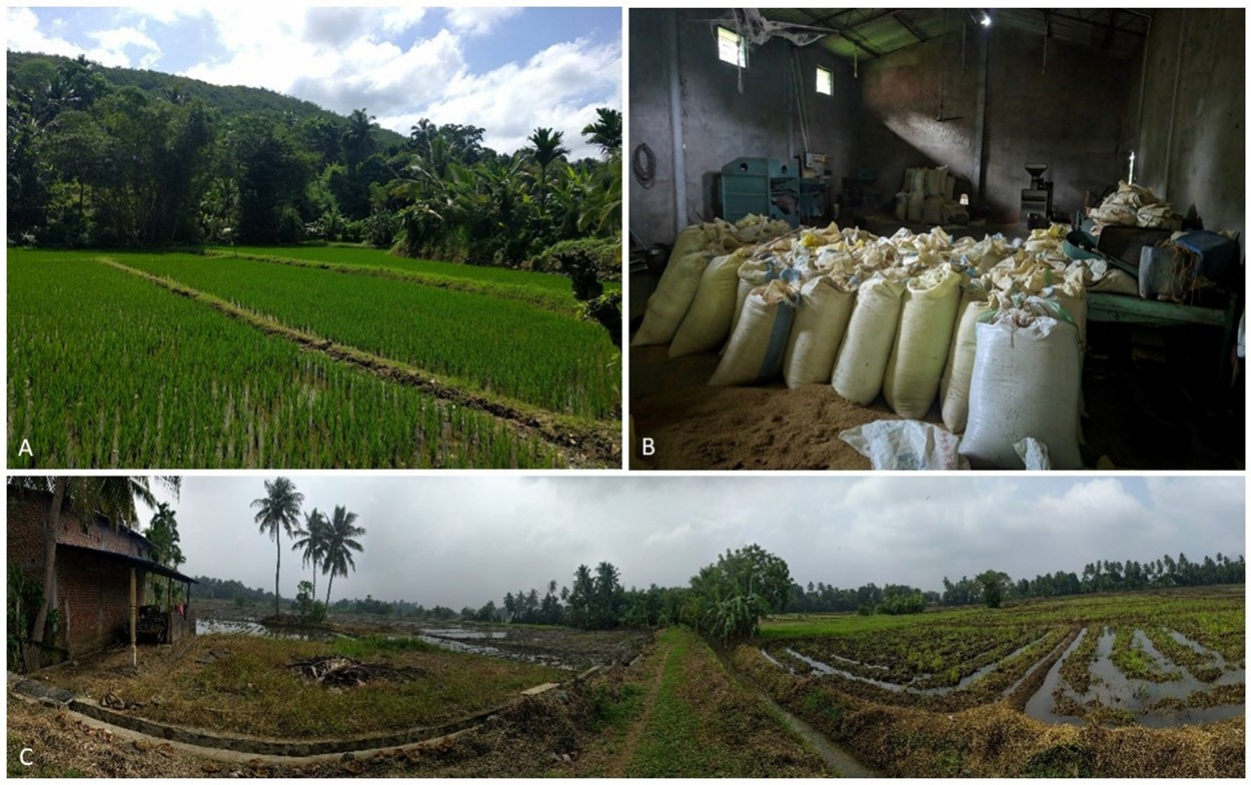
Typical smallholder rice growing environment in Sri Lanka. A. Smallholder rice field embedded in a matrix of surrounding tropical forest. B. Storage facility structure with harvested rice in white storage bags. C. Farmers house and surrounding paddy fields.

The study was conducted between June 2018 and August 2019 in districts within three climatic zones of Sri Lanka: Polonnaruwa (8°01’ N, 81°01’20” E), Kurunegala (7°23’52"N 80°28’10"E) and Gampaha (7°09’13"N 80°08’04"E) (Fig 1). The three districts represent the country’s rainfall gradient, from dry (< 1,750 mm annual rainfall), over intermediate to wet (> 2,500 mm annual rainfall). Total rainfall in mm over the study period, amount of missing days with data and distance were extracted for the nearest station to each sample location from the NOAA climatology network [20]: Dry [874 mm; 62 days missing; 60.6 km], Intermediate [864 mm; 139 days missing; 13.7 km] and Wet [2142 mm; 62 days missing; 40.2 km]. The epidemiology unit of Sri Lanka reported 193, 449 and 193 cases of human leptospirosis during the study period in each district, respectively (http://www.epid.gov.lk/). Sites within districts where chosen to represent the typical smallholder rice growing environments (Fig 2).

### Trapping and tissue collection

Rodents were trapped at smallholder farmer food storage facilities during the dry and wet seasons of 2018 and 2019. In Sri Lanka, there are two cultivation seasons namely Maha and Yala, which are synonymous with two monsoon seasons. Maha season is during “north-east (NE) monsoon” from September to March in the following year. Yala season is during “south-west (SW) monsoon” from May to the end of August. These monsoon periods are referred to as “wet seasons”, the period in between the monsoon seasons is referred to as “dry season”.

Sites were selected ad-hoc within each climate zone. Locations where rodent infestations were common and farmers willing to collaborate were preferred. Ten rat snap traps (Romax Snap R Rat traps) and five mouse snap traps (Romax Snap Mouse trap) were placed in and around 20 storage facilities in the wet and intermediate zone and around ten storage facilities in the dry zone. The differences in sites and trapping effort (see below) was due to the remoteness and hence, less field work activities in the dry zone. Traps were baited with unhulled rice and roasted coconut, set in the evening along potential runways of small mammals and checked the next morning for 2–3 nights until 50 individuals were caught in the dry zone and until 100 individuals were caught in the intermediate and wet zone per year. Trapped rodents were removed, measured, dissected and kidneys stored in 75% alcohol until analyses.

### Small mammal species identification

Rodent species identification was based on well-established external characteristics using body weight (to the nearest g), head-body, tail, hind foot and ear length (to the nearest mm) [21]. In addition, genomic DNA was extracted from 30 mg of kidney tissue with the DNA Mini Kit from MACHEREY-NAGEL. The samples were analysed by a PCR targeting cytochrome b gene (cyt-b, 1140 bp) using primers H15915 (5’-TCT CCA TTT CTG GTT TAC AAG AC-3’) and L14723 (5’-ACC AAT GAC ATG AAA AAT CAT CGT T-3’) following Nicolas et al., 2010. In addition the nuclear gene encoding the interphotoreceptor retinoid binding protein (IRBP, 748 bp) was amplified using primers I1-Rattus (5’-ATTGAGCAGGCTATGAAGAG-3’) and J2-Rattus (5’-TAGGGCTTGCTCYGCAGG-3’) following Pagès et al. 2010. Cytochrome b sequences were finally obtained for 446 individuals and IRBP sequences for 48, allowing molecular-based species identification for 451 of the individuals. For the remaining 49 individuals, we identified species based on the morphometric field data.

Sequences obtained were analysed using Geneious Prime and compared with reference sequences [22–26].

### Molecular detection of pathogenic *Leptospira* spp

DNA was extracted from 25 mg of kidney tissue with the QIAamp DNA Mini Kit (Qiagen, Hilden, Germany). DNA of pathogenic *Leptospira* spp. was detected using a *lipL32*-qPCR previously described by [27] with modifications. The qPCR reaction was performed on a 7500 real-time PCR system (Applied Biosystems, USA) using the PerfeCTa qPCR ToughMix UNG L-ROX Mastermix (QuantaBio, Beverly, USA), 0.5 μM of primer lipL32-45F (5´-AAGCATTACCGCTTGTGGTG-3´) [28] and lipL32-286Rb (5´-GAACTCCCATTTCAGCGAT-3´) [29]), 0.1μM of TaqMan probe lipL32-189P (FAM-5´-AA AGC CAG GAC AAG CGC CG-3´-BHQ) [28], and 5 μL template DNA. Cycling conditions were the following: pre-incubation step at 50°C for 2 min, initial denaturation at 95°C for 10 min, followed by 45 cycles of 95°C for 15 s and 60°C for 60 s. Samples were tested in duplicate. In each run, several non-template controls were included and DNA of *L*. *interrogans* serovar Icterohaemorrhagiae strain RGA corresponding to 10^4^ genome equivalents served as positive control. All samples showing exponential amplification were scored positive for pathogenic leptospiral DNA.

### Molecular characterization and phylogenetic analyses of pathogenic *Leptospira* spp

Species identification in *lipL*-32-positive samples was performed by amplification and sequencing of the *secY* gene [30]. *SecY*-positive samples were further characterized using multilocus sequence typing (MLST, scheme #1) according to Boonsilp et al., 2013 [31].

For phylogenetic analyses, allelic sequences of MLST loci were concatenated in the order of loci used to define the allelic profile to generate a 3111/3112 bp concatemer for each sample. Phylogenetic trees were constructed in MEGA ver. X [32] using the Maximum Likelihood method and Tamura 3-parameter model [33]. Before the analysis, the optimal substitution model for the analyses were identified using the models application in MEGA [34]. The reliability of the tree was assessed by bootstrap analysis with 1000 replicates. For comparison, MLST data from the pubMLST database of human clinical isolates from Sri Lanka and Asia (https://pubmlst.org/) as well as from previously published genome sequences from human clinical isolates of Sri Lanka [35] were included in the analysis.

### Statistical analysis

The presence-absence of pathogenic *Leptospira* spp. in the three climate zones was analyzed by fitting a joint species distribution model with the Hierarchical Modelling of Species Communities (HMSC) package in R [36,37]. HMSC provides hierarchical modelling of species communities with efficient Bayesian parameter estimation [38]. Occurrence probability of pathogenic *Leptospira* spp. was modelled using a probit regression. *Leptospira* species were used as the response variable with the exception of *L*. *kirschneri*, which was rare and omitted from the analysis. Climatic zone, Shannon diversity index and total host abundance at each storage facility were ‘ecological’ explanatory covariates. Shannon diversity was calculated using the simboot package in R [39]. Host species and sex were reservoir variables and season and year were temporal variables. The spatial distance between farms was included as a random effect to account for spatial autocorrelation. Models were fitted in R 4.03 [40] and HMSC version 3.0–9, using default priors [37]. Posterior distributions were sampled with four Markov Chain Monte Carlo chains (MCMC), each of which was run for 75000 iteration. Chains were thinned by 100 and first 25000 iterations removed as a burn-in, yielding 500 posterior samples per chain. MCMC convergence was evaluated using the potential scale reduction factor of model parameters and visual inspection of the chains. Model selection was based on the WAIC criterion [37].

The software SaTScan (v9.6) was used to detect spatial clusters of infected host communities. In brief, the software utilizes circular windows of different sizes to gradually scan across space for increased risk of infection using prevalence as a proxy. Multiple window sizes were used up to circles containing a maximum of 50% of the storage facilities selected in each district [41]. A probability was assigned to each cluster, which determined the risk of infection within a cluster relative to the outside under the null hypothesis of no differences between observed and expected prevalence. The resulting probability was based on 999 Monte Carlo simulations, and the window with the maximum likelihood was the most likely cluster [41].

## Results

### Reservoir community

Over the entire study period, a total of 500 small mammals were collected in the three districts of which 456 were identified as rodent species and 44 as shrews (Table 1, S1 Data). Trapping effort in the wet and intermediate zones to reach the target of 200 individuals was respectively 16 and 15 nights with 300 traps, whereas the trapping effort in the dry zone for 100 individuals was 7 nights with 300 traps.

**Table 1:** Individuals per species per climate zone. In the dry zone, trapping effort was only half that of the trapping effort in the intermediate and wet zone.

Species / Site	Wet zone	Intermediate Zone	Dry Zone
***Rattus rattus***	172	177	89
***Bandicota indica***	9	3	1
***Bandicota bengalensis***	0	1	0
***Mus booduga***	4	0	0
***Suncus murinus***	15	19	10

The rodent and small mammal community in and around storage facilities consisted of four genera representing five species (Table 1). *Rattus rattus* (n = 438, 87.6%) was the most dominant species in all three districts, followed by *Suncus spp*. (n = 44, 8.8%). *Bandicota indica* (n = 13, 2.6%), *Bandicota bengalensis* (n = 1, 0.2%) *and Mus booduga* (n = 4, 0.8%) were rare. The most common species, *R*. *rattus* and *Suncus murinus* were found in all three zones, while *M*. *booduga* was captured in the wet zone only. DNA analysis of mitochondrial and nuclear markers revealed the presence of *S*. *murinus* and *Suncus montanus* cytochrome b haplotypes in our dataset. As all shrews in this study had the morphological characteristics of *S*. *murinus* and were captured in typical *S*. *murinus* environment (peri-domestically) in a low-land area, all *Suncus* spp. in the study were categorized as *S*. *murinus*.

The Shannon diversity of the small mammal community tended to increase from the dry zone (0.39) over the intermediate (0.43) to the wet zone (0.55). Simultaneous comparison of the differences between the Shannon diversity of wet versus dry [p = 0.15], wet versus intermediate [p = 0.27] and intermediate versus dry [p = 0.86] did not result in statistical differences. The Shannon diversity index per storage facility ranged from 0 to 1.13.

### Leptospira species

Three species of human pathogenic *Leptospira* spp. (*L*. *interrogans*, *L*. *borgpetersenii* and *L*. *kirschneri*) were detected in rodent communities in 32 (63%) out of 50 storage facilities. Multiple *Leptospira* species were found in 17 out of 50 storage facilities (34%). Of all 500 small mammals trapped, 61 individuals were infected with pathogenic *Leptospira* spp. (12%). Based on *secY* sequence analysis, the leptospiral species could be identified in 72% of these *lipL32*-positive specimens. *L*. *borgpetersenii* (n = 34, 56%) was most prevalent, followed by *L*. *interrogans* (n = 15, 25%) and *L*. *kirschneri* (n = 1, 2%). Eleven positive samples remained unidentified. Prevalence was highest for *L*. *borgpetersenii* (16%) in the dry climate zone (Table 2, S1 Data) compared to the intermediate and wet zone.

**Table 2:** Observed prevalence of pathogenic *Leptospira* spp. in each climate zone with number of infected individuals indicated in brackets.

Zone (ind.)	*L*. *borgpetersenii*	*L*. *interrogans*	*L*. *kirschneri*	n.d.
**WET (n = 200)**	5% (10)	2% (4)	0.5% (1)	4% (7)
**INTERMEDIATE (n = 200)**	4% (8)	3.5% (7)	0% (0)	1% (2)
**DRY (n = 100)**	16% (16)	4% (4)	0% (0)	1% (2)

**Fig 3:**
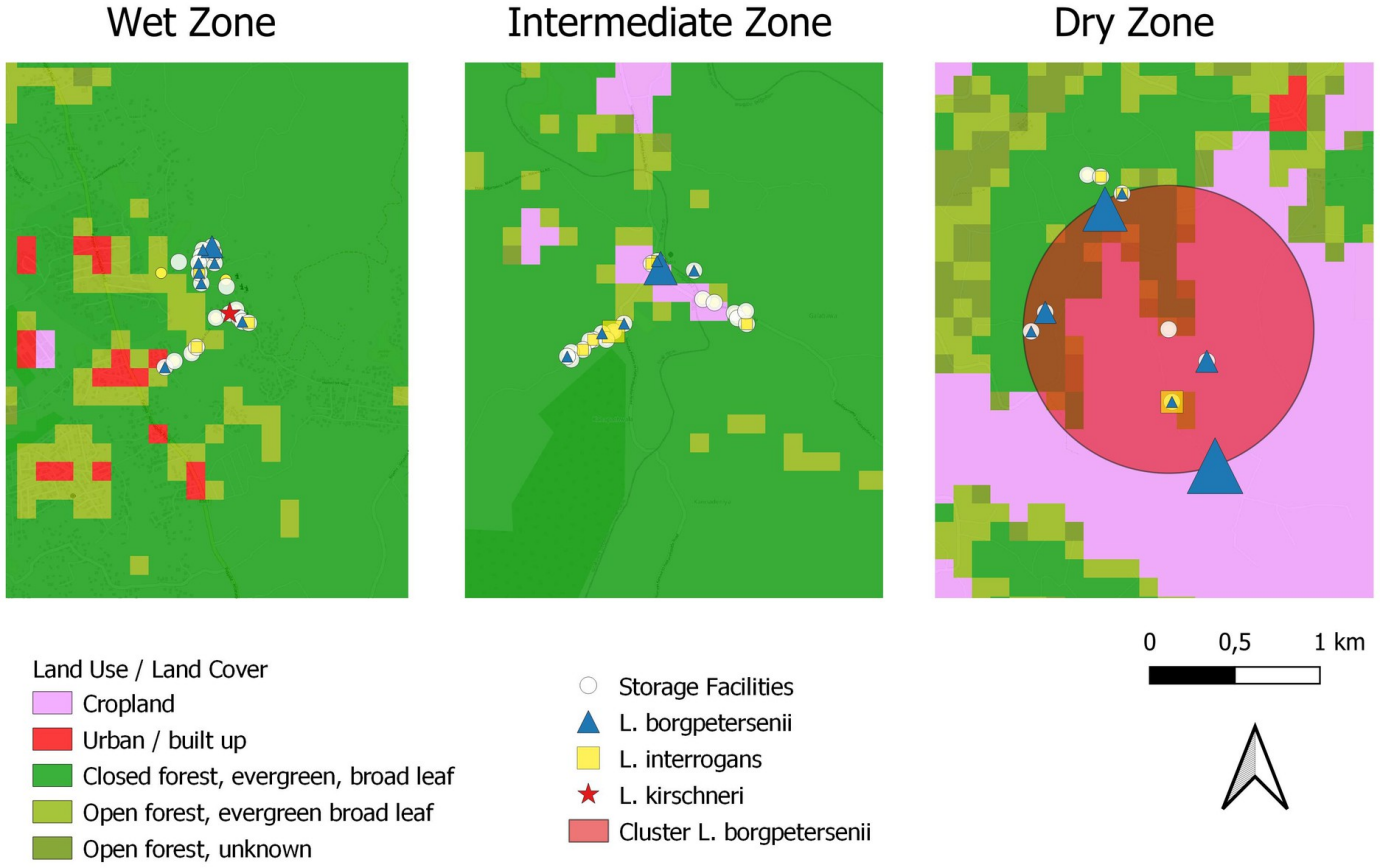
Occurrence of pathogenic *Leptospira* spp. infection in small mammal species at the three climate zones in Sri Lanka. Blue triangles indicate occurrence of *L*. *borgpetersenii*, yellow squares occurrence of *L*. *interrogans* and the red star the occurrence of *L*. *kirschneri*. The size of triangles and squares is proportional to the number of infected animals within storage facility. The large red circle indicates a cluster of increased risk of *L*. *borgpetersenii* infection. Land use and land cover base layer obtained from https://land.copernicus.eu/global/products/lc.

Mapping the occurrence of pathogenic *Leptospira* spp. in small mammal host communities surrounding the storage facilities of smallholder farmers showed that *L*. *borgpetersenii* and *L*. *interrogans* were distributed across the three climate zones, while a single *L*. *kirschneri* was detected in the wet zone only (Table 2, Fig 3). There was no indication of a spatial cluster for *L*. *interrogans*, but a cluster of elevated risk for *L*. *borgpetersenii* infection was located in the dry zone. This cluster consisted of 8 storage facilities accumulating 16 cases of *L*. *borgpetersenii* and having 4.2 times higher risk for infection (p = 0.0012) (Fig 3; red circle).

### Leptospira small mammal reservoir

Pathogenic *Leptospira* spp. were identified in four out of five captured small mammal species (Table 3). *S*. *murinus* was the most important reservoir in terms of *Leptospira* prevalence, while *R*. *rattus* was the only reservoir species harboring both *L*. *borgpetersenii* and *L*. *interrogans*. The single *L*. *kirschneri* was observed in *M*. *booduga*.

**Table 3:** *Leptospira* prevalence in rodent species and shrews present in smallholder food storage facilities in Sri Lanka including pest and conservation status. L. borg is *L*. *borgpetersenii*, L. inter is *L*. *Interrogans*, L. kirsch is *L*. *kirschneri* and L. sp is total *Leptospira* spp. N is sample size, LC—least concern, VU—vulnerable.

Small mammal species (N_total_)	Water affinity^$^	LEPTOSPIRA PREVALENCE [N_infected_]				PEST STATUS^$^		CONSERVATION STATUS^$$^
		L. borg	L. inter	L. kirsch	L. sp*	Rice & Crop fields	Storage & Houses	
Bandicota indica (13)	++	7.7% [1]	0	0	23.1% [3]	✓	-	LC
Bandicota bengalensis (1)	++	0	0	0	0	✓	✓	LC
Rattus rattus (438)	+++	4.6% [20]	3.4% [15]	0	9.8% [43]	✓	✓	LC
Mus booduga (4)	?	0	0	25.0% [1]	0	✓	✓	LC
Suncus spp.** (44)	?	29.5% [13]	0	0	31.8% [14]	-	✓	LC / VU

* Total *Leptospira* samples with 11 which remained unidentified.

** All *Suncus* spp. were categorized as *S*. *murinus*, but DNA analysis of mitochondrial and nuclear markers suggest DNA introgression from the Sri Lankan mountain endemic *S*. *montanus* in these *S*. *murinus* populations.

^$^ Aplin et al., 2003[21] and expert opinion.

^$$^ https://www.iucnredlist.org/

### Joint species distribution model

**Fig 4:**
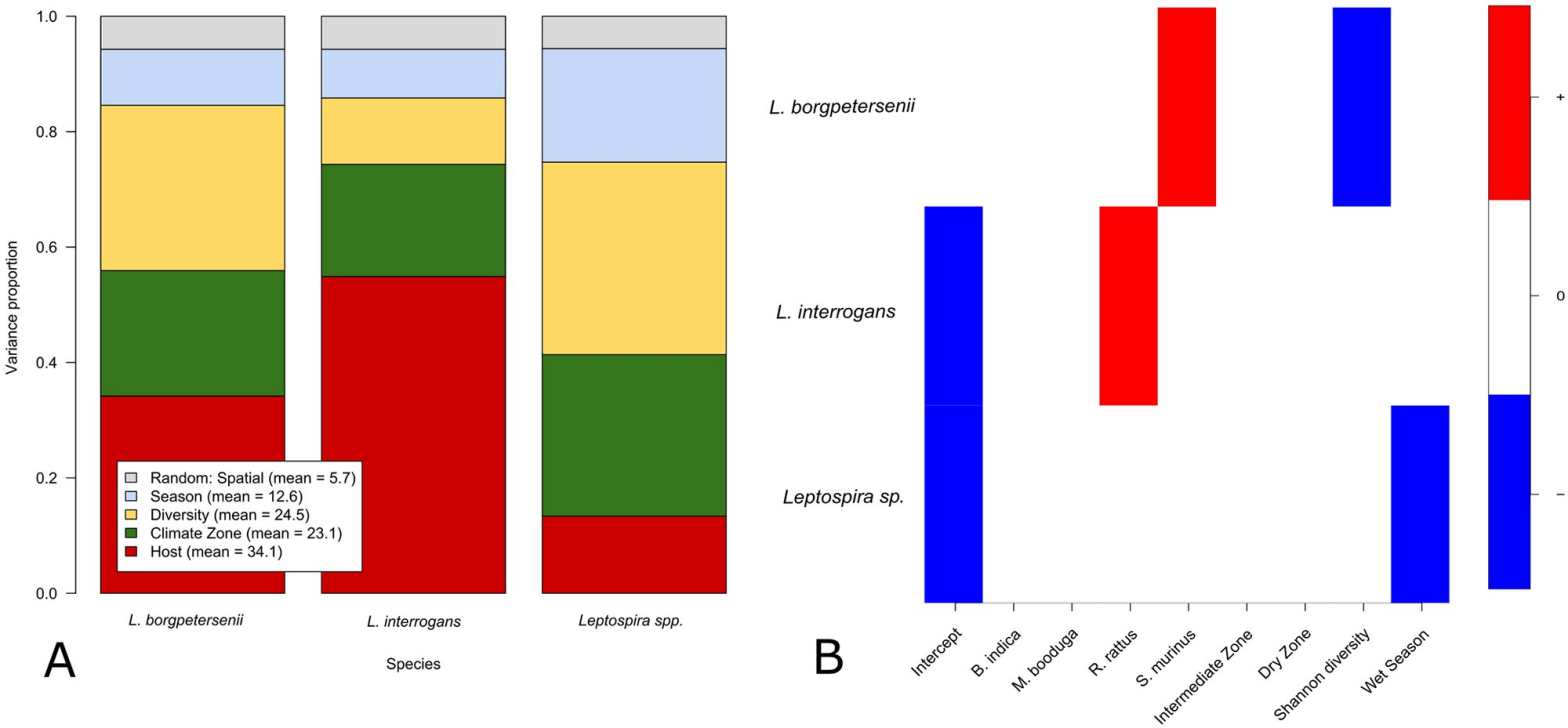
A. Variance partitioning. Variation in *Leptospira* spp. occurrence partitioned into responses to grouped fixed effect for host (species), ecological (climate zone and Shannon diversity), temporal (season) and spatial random effect. B. Parameter estimates and posterior Bayesian support (cutoff probability = 0.9) for either a negative (blue) or positive (red) response.

The final joint species distribution model was superior in terms of WAIC [WAIC = 0.143] compared to the full covariate model [WAIC = 0.151] and an intercept only model [WAIC = 0.159]. Population abundance, annual variation and sex had limited posterior support and were omitted from the analysis. For all 30 parameters the potential scale reduction factor ranged between .999 and 1.008, suggesting that model convergence was reached. The final model revealed a differential response for *L*. *borgpetersenii* and *L*. *interrogans* to environmental and host variables (Fig 4, S2 Data). For *L*. *borgpetersenii*, the Shannon diversity index and the individual host covariate accumulated to 62.8% of the explained variance. For *L*. *interrogans*, the individual host covariate was the dominant explanatory variable, explaining 54.9% of the variation (Fig 4A). Climate zone accounted for about 20% of the explained variance in both *Leptospira* species. For *L*. *borgpetersenii* we found a strong negative association with the Shannon diversity index (Fig 4B—(posterior support = 0.97)) and a moderate positive association with the dry zone (posterior support = 0.87). A positive correlation between the occurrence of *L*. *borgpetersenii* and the individual host covariate for the species *S*. *murinus* (posterior support = 0.91) was also observed. For *L*. *interrogans*, no associations with environmental or temporal covariates were observed, but a positive correlation with *R*. *rattus* individual species (posterior support = 0.91). For the remaining *Leptospira* spp. there was no posterior support for an association with the host covariate represented by individual species, but the directional response of *Leptospira sp*. occurrences with the wet season was negative (posterior support = 0.92).

### Phylogenetic analyses based on concatenated MLST allele sequences

**Fig 5:**
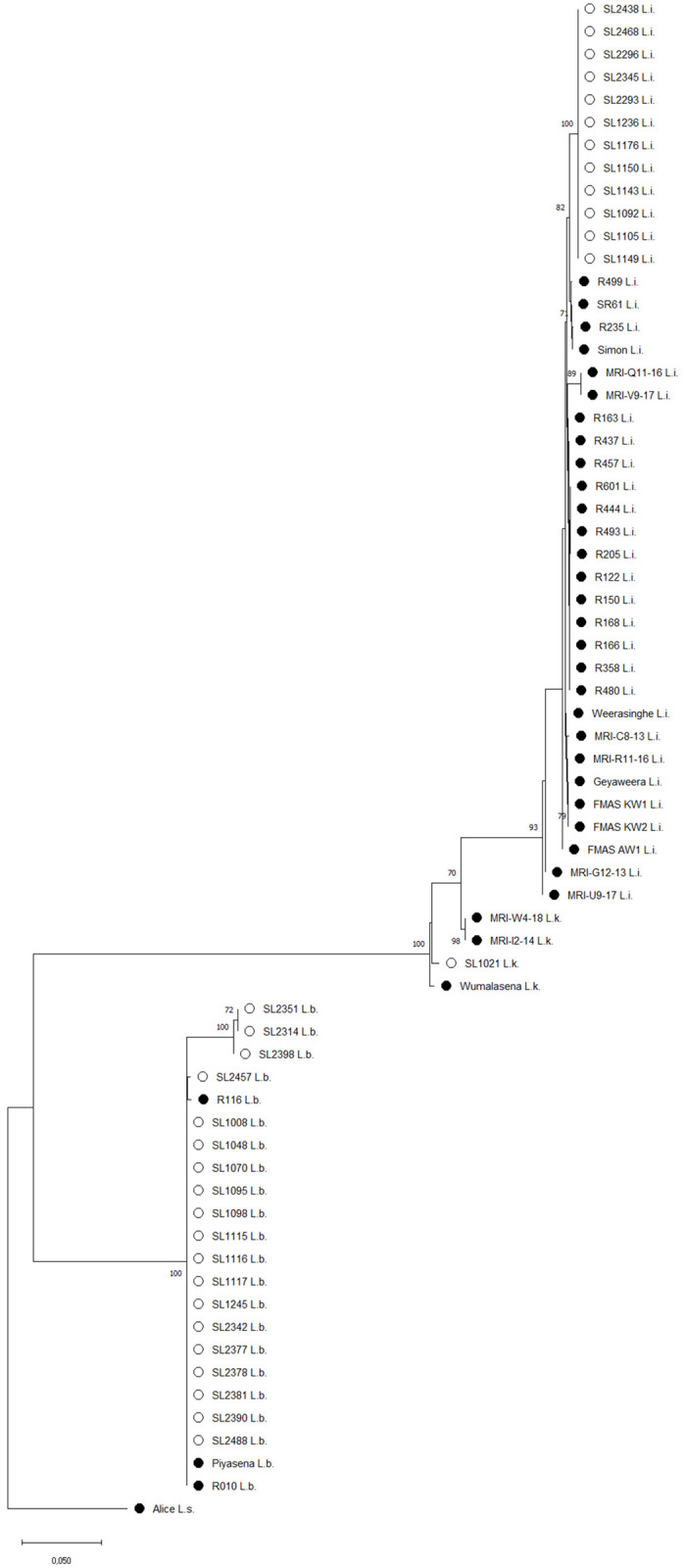
Evolutionary analysis of concatenated MLST allele sequences The tree was inferred in MEGA X using the Maximum Likelihood method and Tamura 3-parameter model. Bootstrap values above 70% are shown next to the branches. MLST sequences from *Leptospira interrogans* (L.i.), *Leptospira borgpetersenii* (L.b.) and *Leptospira kirschneri* (L.k.) obtained from specimen collected in this study are shown (○). For comparison, MLST sequences from human clinical isolates originating from Sri Lanka were included in the analysis (●). The tree was rooted using concatenated MLST sequences of *Leptospira santarosai* (L.s.) strain Alice.

Further characterization of pathogenic *Leptospira* species using MLST was successful in 53% (n = 32) of *lipL32*-positive samples and identified four new sequence types (ST294, ST323, ST325 and ST326). *L*. *borgpetersenii* positive samples showed the highest genetic diversity with four different sequence types (ST144, ST323, ST325, and ST326). Two clonal complexes were defined at the single-locus variant level (ST144-ST325 and ST323-ST326). Phylogenetic analyses based on the concatenated MLST allele sequences observed in this study together with published sequence data from human clinical isolates from Sri Lanka confirmed that rodents and small mammals act as a reservoir for human leptospirosis. All samples positive for *L*. *borgpetersenii* grouped into two clusters, with one cluster containing the majority of samples together with human clinical strains from Sri Lanka (Fig 5). The majority of *L*. *borgpetersenii* positive samples belonged to sequence type ST144, which has already been isolated from four human clinical cases in Sri Lanka (strain R010, region Gampaha and strain Piyasena, region unknown), Laos and Thailand between 1964 and 2007, showing its potential to cause human infection. Moreover, *L*. *borgpetersenii* ST144 was found in all three climate zones of Sri Lanka and showed a similar distribution in *R*. *rattus* and *S*. *murinus* (Table 4). Other STs of *L*. *borgpetersenii* (ST323, ST325 and ST326) have been found rarely and exclusively in *R*. *rattus*. In this study, *R*. *rattus* harbored the highest variety of pathogenic *Leptospira* species (*L*. *borgpetersenii* and *L*. *interrogans*) and MLST types (all four STs of *L*. *borgpetersenii*).

**Table 4:** Occurrence of *Leptospira* MLST sequence types in each small mammal species and climate zone. n is the number of samples with specific ST in the respective category, N is the total number of samples with specific ST.

		Distribution of *Leptospira* MLST sequence types (ST) (n)					
		*L*. *borgpetersenii*				*L*. *interrogans*	*L*. *kirschneri*
		ST144 (N = 15)	ST323 (N = 2)	ST325 (N = 1)	ST326 (N = 1)	ST294 (N = 12)	ST70 (N = 1)
Small mammal species	***Rattus rattus***	8	2	1	1	12	0
	***Suncus murinus***	7	0	0	0	0	0
	***Mus booduga***	0	0	0	0	0	1
Zone	**WET**	4	1	0	0	3	1
	**INTERMEDIATE**	3	1	0	0	6	0
	**DRY**	8	0	1	1	3	0

Sequence type ST294 was described for the first time in this study and interestingly, was identified in all typeable *L*. *interrogans* positive samples forming a distinct cluster separated from *L*. *interrogans* clinical strains from Sri Lanka (Fig 5) and Asia. Remarkably, *L*. *interrogans* ST294 was observed across all three Sri Lankan climate zones and was exclusively found in *R*. *rattus*, implying a specific host adaptation of this sequence type to *R*. *rattus*.

*L*. *kirschneri* ST70 observed in one infected *M*. *booduga* specimen was previously described in bats and mice from Indonesia and China but seems relatively closely related to another clinical isolate from a human leptospirosis case in Sri Lanka based on the phylogenetic tree (Fig 5).

## Discussion

Leptospirosis is a notifiable disease in Sri Lanka illustrating its public health importance. A recent review suggests a gross underestimation of the true leptospirosis burden in the country, with an estimate for hospitalization rates of 300 per 100,000 (Warnasekara et al., 2019 [42]). The tropical climate conditions in Sri Lanka generally favour outbreaks of this disease [43]. While human cases occur throughout the year, outbreaks of leptospirosis have been coinciding with the rice cultivation seasons [18]. Paddy farming or exposure to agricultural water sources alongside exposure to ditch, canal or domestic sewer water have all been identified as important sources of human infection in Sri Lanka [18]. Rodent populations, cattle and domestic mammals play an important role in persistent excretion and transmission of leptospires and both wildlife and domestic reservoirs can introduce pathogens from paddy fields to farmers’ houses.

In this study, small mammal populations in and around food storage facilities were sampled. Rodent populations acting as disease reservoirs pose not only a threat to public health, they can also cause considerable damage to stored food (Brown et al., 2017 [44]; Jacob & Buckle, 2018 [45]) including in Sri Lanka [19]. Hence, storage structures seem an important interface for rodent-human contact where associated problems concentrate. Building a knowledge base on those species that concern both food security and public health can help optimise management strategies that focus on the most relevant species.

Out of the 500 small mammals collected the black rat (*R*. *rattus*) was the most common species, followed by the Asian house shrew (*S*. *murinus*). The Asian house shrew is a commensal species found in the proximity of houses and storage facilities. While this species is mainly insectivorous, damage to stored food by this species is common [46]. *S*. *murinus* have also been identified as a non-rodent host for plague transmission, illustrating its potential as a threat to public health [47]. Notably, the cytochrome-b sequence data of the *Suncus* shrews indicated that, in addition to the Asian house shrew (*S*. *murinus*), the Asian highland shrew (*S*. *montanus*) may also be present in our samples. As was shown previously [24,26], hybridization between *S*. *montanus* and *S*. *murinus* and introgression of *S*. *montanus* alleles into *S*. *murinus* may occur and we suspect this phenomenon to explain the presence of *S*. *montanus* cytochrome-b haplotypes in our data. Sri Lanka has an enormous small mammal biodiversity and several species are endemic and endangered amongst which seven belong to the shrew family, such as the Sri Lankan long-tailed shrew (*Crocidura miya*), and the jungle shrew (*Suncus zeylanicus)* [48]. *S*. *montanus* is categorized as a vulnerable species. The taxonomic status of these *Suncus* samples and the complex relationship between the widespread Asian house shrew and the highly endemic Asian highland shrew warrants further investigation, especially regarding any management activities, be it for public health or food security. The black rat is a notorious pest species in both crop fields and in commensal situations. With a movement radius of several hectares in rice field habitat [49], this species could be key in seasonally transporting pathogens between paddy fields into storage facilities and houses through their regular movements among these habitats [50].

In this study, three pathogenic *Leptospira* species, *L*. *interrogans*, *L*. *borgpetersenii* and *L*. *kirschneri* were identified. Our MLST and phylogenetic analyses show that rodents and small mammals sampled across all three climate zones in Sri Lanka were infected with *Leptospira* strains strongly related to clinical strains previously isolated from human leptospirosis cases in Sri Lanka, implying their important role as reservoir for human infection. For example, *R*. *rattus* and *S*. *murinus* both harbored *L*. *borgpetersenii* ST144 that was observed in all climate zones and had been isolated from clinical samples in Sri Lanka previously. Albeit the Black rat showed the highest diversity of different *Leptospira* species and MLST sequence types, all *L*. *interrogans* positive samples belonged to the same newly described sequence type ST294 and were exclusively found in *R*. *rattus*, implying a specific host adaptation of this sequence type to *R*. *rattus*. *L*. *borgpetersenii* was most prevalent in the host community samples and the only one detected in different rodent and small mammal species. During meat inspection in Colombo, Sri Lanka, the same *Leptospira* species was the most frequently found in cattle kidney samples [15]. *L*. *interrogans*, was the second most prevalent *Leptospira* species detected and found only in the black rat (*R*. *rattus*), while the remaining *L*. *kirschneri* species was detected in *M*. *booduga*. This suggests that the small mammal reservoir can maintain *Leptospira* populations and possibly enhance its spread. *L*. *borgpetersenii* has a reduced ability to survive outside the host animals and it is hypothesized that the mode of transmission for this particular spirochaete bacteria relies more on host-host interactions, while *L*. *interrogans* can persist in aquatic environments until a new host arrives allowing for indirect host-environment-host transmission [51–53]. The results from our field study, with *L*. *borgpetersenii* detected in multiple host species, and *L*. *interrogans* found in a single species known for its affinity with water seem to confirm that hypothesis.

The findings from field work regarding rodent species distribution, abundance and presence of leptospires seem robust for descriptive purpose despite the bias in sampling design that resulted in more intense trapping and therefor more individuals sampled in the wet and intermediate versus the dry zone. Potential bias was mitigated by using the same number of individuals per site relative to trapping effort. The use of storage facilities for sampling was based on a similar selection process in all climatic zones to cover a representative portion in an attempt to capture variability regarding size of farms and surrounding habitat.

The main limitation of this study was lack of repeated sampling within each climate zone. Nonetheless, the statistical model suggests that the probability of *L*. *borgpetersenii* occurrence is inversely correlated with the small mammal diversity around the storage facilities, while it was positively associated with the presence of *S*. *murinus*. For *L*. *interrogans* the presence of *R*. *rattus*, being an exclusive host in this study for *L*. *interrogans*, was the dominant explanatory factor. 93% of all *Leptospira* spp. infections were detected in either the Asian house shrews (*S*. *murinus*) or the black rat (*R*. *rattus*). This outcome suggests that, irrespective of climate zone, further research would benefit from focusing on those particular species. Investigating how targeted pest management actions, while safeguarding the others, could influence both food security and the probability of *Leptospira* spill-over to both livestock and humans, appears promising.

## Conclusion

Building a knowledge base on those small mammal species that pose a threat to food security and health, be it livestock or humans, is necessary to help to optimize mitigation strategies. Notably in Sri Lanka, which is considered a biodiversity hotspot, small mammal pest management strategies need to overcome the challenge of accounting for rare and endemic species present in the area. Listing those that pose threats to food or health can enable choosing alternative mitigation strategies and the data presented here can support such activities. Based on this descriptive study, further longitudinal studies should be conducted to consider areas and times of varying rodent abundance as this study concentrated on situations with high rodent abundance and consequent pathogen prevalence. In addition, including further *Leptospira* hosts such as livestock and human data seems appropriate for obtaining a more holisitic pattern of the role of small mammals in cropping and pathogen transmission.

## Supporting information

S1 Data The data on *Leptospira* species, host community and additional variables. (XLSX)

S2 Data The beta and variance estimates for Fig 4. (XLSX)
